# Imaging biomarker associated with early neurological deterioration in isolated pontine infarction

**DOI:** 10.3389/fneur.2024.1492166

**Published:** 2024-12-19

**Authors:** Xiaowei Song, Feifei He, Duoduo Hou, Hongliang Zhao, Chenming Wei, Le Chen, Zhuoma Pengmao, Jian Wu

**Affiliations:** ^1^Department of Neurology, Beijing Tsinghua Changgung Hospital, School of Clinical Medicine, Tsinghua University, Beijing, China; ^2^Department of Neurology, Beijing Geriatric Hospital, Beijing, China; ^3^Department of Radiology, Beijing Tsinghua Changgung Hospital, School of Clinical Medicine, Tsinghua University, Beijing, China; ^4^IDG/McGovern Institute for Brain Research, Tsinghua University, Beijing, China

**Keywords:** ischemic stroke, isolated pontine infarction, early neurological deterioration, imaging biomarker, cerebral small vessel disease

## Abstract

**Objective:**

To investigate the association between cerebral small vessel disease burden, along with its individual imaging features, as well as other imaging features and early neurological deterioration in isolated pontine infarction.

**Methods:**

107 patients with acute isolated pontine infarcts, within 24 h of symptom onset, were retrospectively analyzed. The mean age of the participants was 67 years. The burden of cerebral small vessel disease on brain MRI was assessed, including white matter hyperintensities (WMH), lacunes, cerebral microbleeds (CMB), and enlarged perivascular spaces (EPVS) for each patient. Additionally, other imaging biomarkers including basilar artery plaque features on high-resolution MR vessel wall imaging and intracranial artery stenosis were evaluated simutaneously. END was defined as an increase of ≥1 point on the motor component of the National Institutes of Health Stroke Scale (NIHSS) or an increase of ≥2 points on the total NIHSS score within 72 h of admission. Statistical analyses were performed using t-tests, chi-square tests, and logistic regression.

**Results:**

33.6% (36/107) of the patients experienced END. The END group exhibited a higher prevalence of hyperlipidemia compared to the non-END group (66.7% vs. 43.7%, *p* = 0.024). Over 50% of the mechanisms of infarction can be attributed to basilar artery branch disease in both groups, with 58.3% in the END group and 50.7% in the non-END group. In a multivariate regression analysis, neither the total burden of cerebral small vessel disease nor the presence of WMH, CMB, lacunes, or EPVS were significantly associated with END (all *p* > 0.05). However, basilar artery intraplaque hemorrhage observed on vessel wall imaging was independently associated with END (OR = 3.233, 95% CI 1.032–10.123, *p* = 0.044).

**Conclusion:**

No association was found between the imaging features of cerebral small vessel disease and END in patients with isolated pontine infarction. However, basilar artery intraplaque hemorrhage serve as an independent predictor of END in this population.

## Introduction

Isolated pontine infarction is the most prevalent type of posterior circulation stroke, with approximately one-third of patients experiencing early neurological deterioration (END) (1), Those with END typically have a poor prognosis (2). The underlying mechanisms of isolated pontine infarction can be categorized into three types: basilar artery branch disease (BABD), small-artery disease (SAD) and large-artery occlusive disease (LAOD) (3). Identifying the predictors of END in these patients is crucial for risk assessment and the development of targeted interventions. Each type can be reflected by some imaging features, such as the presence of basilar artery vulnerable plaque, degree of stenosis, etc.

Cerebral small vessel disease (CSVD) is a complex clinical syndrome characterized by the presence of lacunes, white matter hyperintensities (WMH), enlarged perivascular spaces (EPVS), microbleeds (CMB), and brain atrophy observed on brain imaging (4). The burden of CSVD has been identified as a predictor of stroke outcomes in previous studies (5). One study indicated that a higher CSVD score is associated with poor prognosis and recurrence in acute ischemic stroke (6). Conversely, another study involving patients with mild ischemic stroke found that imaging markers of CSVD did not correlate with END (7). Additionally, a small sample study examined the relationship between WMH and END in patients with isolated pontine infarction, revealing that both periventricular and subcortical WMH were useful in predicting END in this population (8). However, this study focused solely on WMH, neglecting other features of CSVD, including lacunes, CMB, and EPVS. CSVD may adversely affect stroke prognosis by compromising dynamic cerebral autoregulation (9), which is also one of the contributing factors of END.

Therefore, imaging biomarker in predicting END in patients with isolated pontine infarction need further study, especially the correlation between the CSVD burden, along with its individual features and END requires comprehensively investigation. The objective of this study was to examine the association between CSVD burden, along with its individual imaging features, and other imaging biomarkers and END in patients with isolated pontine infarction.

## Methods

**Figure 1:**
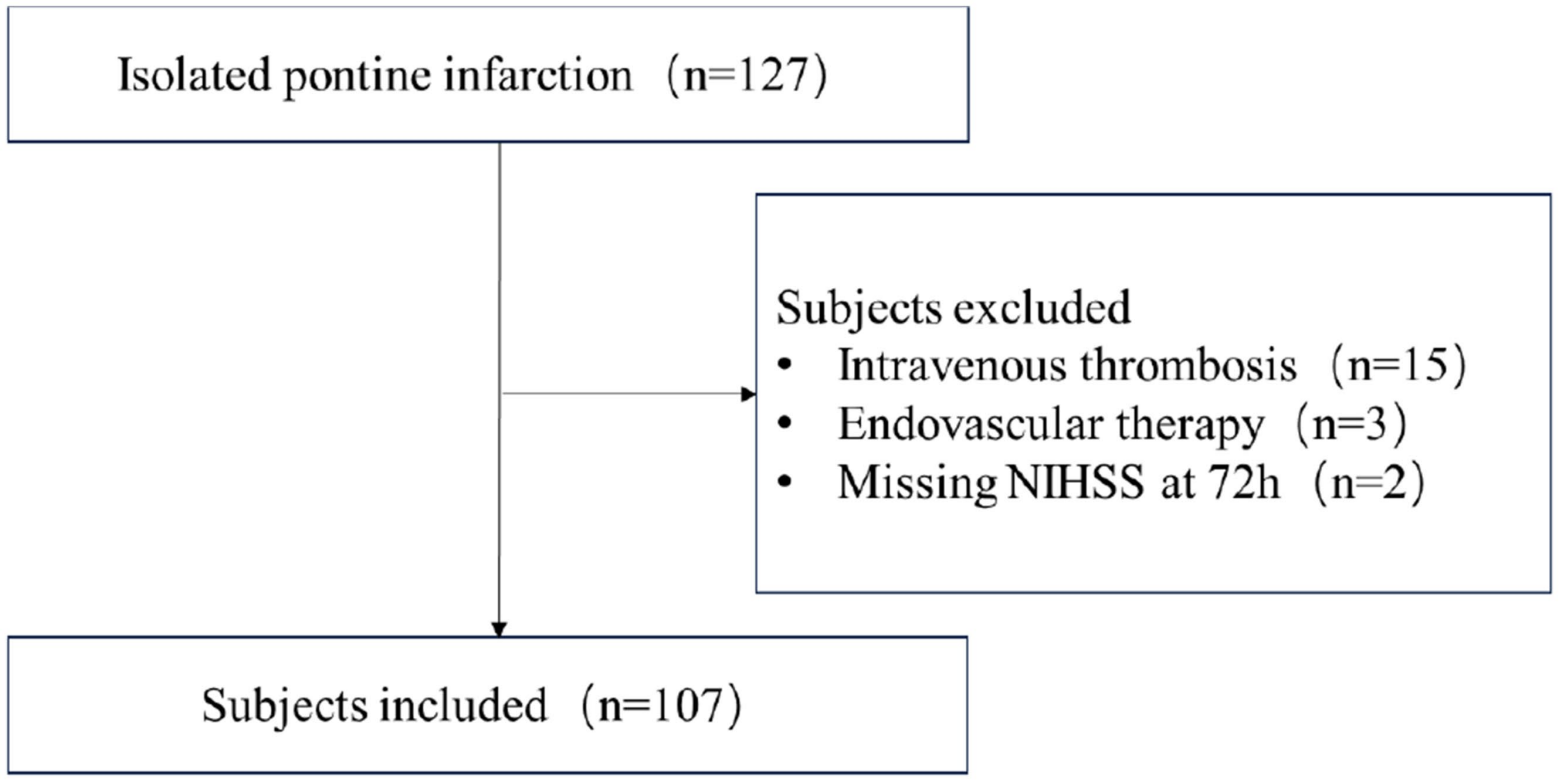
Flow chart of subjects screening.

This is a retrospective study conducted at a comprehensive stroke center. Ischemic stroke patients with isolated pontine infarction from January 2020 to December 2022 were screened independently by two neurologists. Subjects were included if they met the following criteria: (1) age ≥ 18 years; (2) presentation with focal neurological symptoms and corresponding pontine infarction validated by Diffusion Weighted Imaging (DWI); (3) admission within 24 h of symptom onset; (4) patients with complete imaging evaluations, including MRA/CTA and intracranial vessel wall imaging. Subjects were excluded if they had received intravenous thrombolysis, underwent endovascular thrombectomy, or had missing recordings of neurological changes or NIHSS after admission. The flow-chart of subjects screening was shown in Figure 1.

Demographic characteristics, atherosclerotic risk factors (such as hypertension, diabetes, hyperlipidemia, previous stroke history, coronary artery disease, and chronic kidney disease), blood test results, blood pressure at admission, and the National Institutes of Health Stroke Scale (NIHSS) scores at admission and after deterioration were all obtained from the medical report. The infarct mechanism was categorized as basilar artery branch disease (BABD), small-artery disease (SAD) and large-artery occlusive disease (LAOD) based on the infarct location and vascular lesions (3). In this study, END was defined as an increase of ≥1 point on the motor NIHSS or ≥2 points on the total NIHSS score within 72 h of admission (8).

This study protocol was approved by Ethics Committee of Beijing Tsinghua Changgung Hospital (22028-0-02), and informed consent was waived due to the retrospective nature of the cohort.

### Imaging protocol

All patients were scanned using a 3.0 T MR scanner (Discovery 750, GE Healthcare, Milwaukee, USA) equipped with an 8-channel head coil. The protocol included brain MRI followed by MRA. The detailed sequences and their parameters were as follows: (1) T1-weighted (T1W) fast spin echo (FSE), TR/TE 1750/24 msec, field of view (FOV) 24 cm × 24 cm, matrix 320 × 256, thickness 5 mm; (2) T2-weighted (T2W) FSE, TR/TE 5000/95 msec, FOV 24 cm × 24 cm, matrix 512 × 512, thickness 5 mm; (3) T2 fluid-attenuated inversion recovery (FLAIR), FSE, TR/TE 9000/145 msec, FOV 24 cm × 24 cm, matrix 256 × 256, thickness 5 mm; (4) diffusion-weighted imaging (DWI), echo-planar imaging (EPI), TR/TE 3000/65 msec, FOV 24 cm × 24 cm, matrix 160 × 160, thickness 5 mm; (5) apparent diffusion coefficient (ADC), EPI, TR/TE 3000/65.7–84; (6) susceptibility-weighted imaging (SWI), gradient echo (GRE), TR/TE 80–15,000/45.

MRA included 3D time-of-flight (TOF) MRA and Cube T1-weighted imaging. The 3D TOF MRA was obtained using an axial fast-spin echo (FSE) sequence with the following parameters: TR/TE of 22/2.5 msec, a field of view (FOV) of 22 × 21 cm, a flip angle (FA) of 20°, and a spatial resolution of 0.6 × 1.0 × 1.2 mm^3^. The 3D Cube T1-weighted imaging utilized a spin echo (SE) sequence with parameters of TR/TE of 800/16 msec, a FOV of 23 × 18 cm, an FA of 90°, and a spatial resolution of 0.7 × 0.6 × 0.6 mm^3^.

### Image analysis

All images were evaluated by two experienced radiologists who were blinded to the clinical information. A new pontine infarct was defined as a hyperintense area on DWI with a corresponding decrease in signal on the ADC.

Cerebral small vessel disease burden was evaluated by two independent neurologists according to previously established criteria, which has been established to have quite good consistency among evaluators and comparability with quantitative study (10). The total CSVD score were calculated based on the following features: 1 point for each of the following: ≥1 lacune, deep WMH (dWMH) ≥ 2, periventricular WMH (pWMH) = 3, ≥ 1 CMB, and moderate to severe (2–4) PVS. Lacunes were defined as rounded or ovoid lesions measuring 3–20 mm in diameter, located in the basal ganglia, internal capsule, centrum semiovale, or brainstem, exhibiting cerebrospinal fluid (CSF) signal intensity on T2-weighted and FLAIR images, typically with a hyperintense rim on FLAIR and no increased signal on DWI. CMBs were evaluated using SWI and defined as small (≤5 mm), homogeneous, round foci of low signal intensity on gradient echo images in the cerebellum, brainstem, basal ganglia, white matter, or cortico-subcortical junction, differentiated from vessel flow voids and mineral deposits in the globus pallidus. Both deep and periventricular WMH were assessed according to the Fazekas scale, which ranges from 0 to 3 (11). PVS was defined as small (≤1 mm) punctate hyperintensities (if perpendicular) and linear hyperintensities (if longitudinal to the plane of the scan) on T2-weighted images in the basal ganglia or centrum semiovale, and they were rated on a previously described, validated semiquantitative scale ranging from 0 to 4.

In addition, intracranial artery stenosis was recorded in each vessel, with the degree of stenosis categorized as <30%, 30–49%, 50–69%. Intraplaque hemorrhage (IPH) on basilar artery was identified on Cube T1-weighted imaging, defined as T1-hyperintense plaque exhibiting signal intensity greater than that of the surrounding brain parenchyma or muscle (12).

### Statistical analysis

SPSS 19.0 was utilized for statistical analysis. Variables with a normal distribution were presented as mean ± standard deviation (SD), while those with a skewed distribution were presented as median (interquartile range, IQR). T-tests and chi-square tests were employed to compare variables between the END and non-END groups. Multivariate logistic regression was conducted to adjust for confounding factors with a *p*-value of less than 0.1 in univariate analysis and to identify the association between cerebral small vessel disease burden and END in these patients. A statistical significance level was set at 0.05.

## Results

A total of 107 subjects who met the criteria were included in this study. The mean age of the participants was 67 years, with a male predominance of 77.6% (83 out of 107). Among these subjects, 33.6% (36 out of 107) experienced early neurological deterioration (END) within 72 h of admission, with most cases occurring within 48 h. A comparison of demographic and risk factors between the END and non-END groups is presented in Table 1. Patients with END exhibited a higher prevalence of hyperlipidemia, and a greater proportion of patients in the END group were on dual antiplatelet therapy. The mechanisms of infarction differed significantly between the END and non-END groups; Both groups had a contribution of large artery atherosclerosis (LAA) exceeding 50%, while small artery disease (SAD) was more prevalent in the non-END group. No statistically significant differences were found in other atherosclerotic risk factors between the END and non-END groups.

**Table 1:** Baseline characteristics of subjects between END and non-END group.

Variables M ± SD or *n* (%)	END group (*n* = 36)	Non-END group (*n* = 71)	*p*-value
Age, years	64.86 ± 11.34	68.62 ± 10.05	0.083
Male	31 (86.1)	52 (73.2)	0.132
Hypertension	30 (83.3)	51 (71.8)	0.190
Diabetes	21 (58.3)	49 (69.0)	0.272
Hyperlipidemia	24 (66.7)	31 (43.7)	0.024
CAD	6 (16.7)	17 (24.3)	0.367
CKD	1 (2.9)	10 (14.3)	0.143
Previous stroke	15 (41.7)	34 (47.9)	0.542
SBP	155.5 ± 15.3	154.2 ± 21.1	0.725
DBP	83.9 ± 8.7	83.4 ± 14.4	0.841
Glucose (mmol/L)	8.89 ± 3.46	8.53 ± 3.44	0.620
HbA1C (%)	7.39 ± 1.92	7.02 ± 1.65	0.337
TC (mmol/L)	4.52 ± 1.01	4.30 ± 1.00	0.322
TG (mmol/L)	1.77 ± 1.10	1.58 ± 0.83	0.343
HDL-C (mmol/L)	0.98 ± 0.23	1.07 ± 0.27	0.106
LDL-C (mmol/L)	2.88 ± 0.78	2.57 ± 0.88	0.093
Intensive statin	3 (8.3)	3 (4.2)	0.669
Dual-antiplatelet	23 (63.9)	26 (36.6)	0.007
NIHSS at admission^*^	2 (1,4)	2 (1,3)	0.598
Infarct mechanism			0.003
BABD	21 (58.3)	35 (50.7)	
SAD	8 (22.2)	32 (46.4)	
LAOD	7 (19.4)	2 (2.9)	

CAD, coronary artery disease; CKD, chronic kidney disease; SBP, systolic blood pressure; DBP, diastolic blood pressure; TC, total cholesterol; TG, triglycerides; HDL-C, high-density lipoprotein cholesterol; LDL-C, low-density lipoprotein cholesterol; BABD, basilar artery branch disease; SAD, small-artery disease; LAOD, large-artery occlusive disease.

* M [IQR], Mann–Whitney test.

As shown in Table 2, the features of cerebral small vessel disease, including lacunes, white matter hyperintensities, microbleeds, and enlarged perivascular spaces (EPVS), were compared between the END and non-END groups. However, neither the total burden of cerebral small vessel disease (CSVD) nor its individual features demonstrated any significant differences between the END and non-END groups (all *p* > 0.05). Additionally, the prevalence of basilar artery (BA) stenosis, internal carotid artery (ICA) and middle cerebral artery (MCA) stenosis, and basilar artery intraplaque hemorrhage (IPH) were also compared between the two groups. The results indicated that patients with END were more likely to have basilar artery IPH.

**Table 2:** Image features comparison between END and non-END group.

Variables	END group (*n* = 36)	Non-END group (*n* = 71)	*p*-value
Total CSVD scale	1 (0,2)	2 (1,2)	0.157
Lacune	22 (61.1)	55 (77.5)	0.075
WMH-total score	2 (2,4)	3 (2,4)	0.093
pWMH	2 (1,2)	2 (1,3)	0.057
dWMH	1 (0,2)	1 (1,2)	0.282
CMB	11 (30.6)	25 (35.2)	0.630
EPVS	4 (11.1)	8 (11.3)	1.000
BA ≥ 50% stenosis	16 (44.4)	25 (35.7)	0.382
ICA/MCA ≥ 50% stenosis	17 (48.6)	20 (30.3)	0.070
Basilar artery IPH	10 (31.3)	8 (12.3)	0.024

WMH, white matter hyperintensity; pWMH, periventricular WMH; dWMH, deep WMH; EPVS, enlarged perivascular spaces; BA, basilar artery; ICA, internal carotid artery; MCA, middle cerebral artery; IPH, intraplaque hemorrhage.

In multivariate regression analysis, after adjusting for confounding variables, we did not observe any significant association between CSVD burden and END, nor any relationship between CSVD features and END, with all *p*-values exceeding 0.05. However, basilar artery IPH was independently associated with END in the multivariate regression analysis, with odds ratio (OR) = 3.233 and a 95% confidence interval (CI) of (1.032, 10.123; Table 3).

**Table 3:** Predictors of END in logistic regression analysis.

	Multivariate regression		
	OR	95% CI	*p*-value
Total CSVD scale	0.881	0.585–1.327	0.544
Lacune	0.596	0.231–1.535	0.283
WMH-total score	0.876	0.624–1.230	0.444
pWMH	0.736	0.420–1.290	0.284
dWMH	0.946	0.547–1.636	0.842
CMB	0.976	0.395–2.411	0.959
EPVS	1.029	0.275–3.847	0.966
BA ≥50% stenosis	1.711	0.694–4.219	0.244
ICA/MCA ≥50% stenosis	2.319	0.928–5.797	0.072
Basilar artery IPH	3.233	1.032–10.123	0.044

Adjusting for age, hyperlipidemia in multivariate regression analysis.

## Discussion

Our study demonstrates negative correlations between the burden of cerebral small vessel disease and early neurological deterioration (END) in patients with isolated pontine infarction. However, intraplaque hemorrhage (IPH) in the basilar artery is independently correlated with END in these patients. The findings of our study could enhance our understanding of END in isolated pontine infarction.

### Prevalence of END in isolated pontine infarction and underlying mechanism

One-third of the patients in our study presented with END, and most cases of END occurred within 48 h after the onset of symptoms. Patients with isolated pontine infarction may initially present with dizziness and limb numbness, but they typically have a lower NIHSS score. In many instances, the dizziness is non-specific and challenging to differentiate from other causes. The most common mechanism of isolated pontine infarction identified in our study is basilar artery branch disease, which aligns with findings from previous study. In that study, BABD was the most frequent cause of isolated pontine ischemia, followed by SAD and LAOD (3). These results suggest that patients experiencing sudden dizziness or other mild posterior circulation symptoms warrant increased attention, particularly in the initial days, as they are more likely to experience symptom deterioration. Furthermore, other studies have indicated that patients with pontine infarction induced by branch atheromatous disease are more likely to experience recurrence at the same site and through the same mechanism (13). Therefore, rapid evaluation of the etiology and mechanism is essential, as it will provide valuable evidence for optimal medical management in this patient population.

### CSVD and END in isolated pontine infarction

The association between CSVD and stroke outcomes has been extensively studied, yet the findings remain inconclusive (6, 14–16). While the predictive value of CSVD in early neurological deterioration has not been thoroughly investigated, a study involving 82 patients indicated that severe WMH are associated with END in cases of isolated pontine infarction, both severe pWMH (OR = 6.17; 95% CI 1.93–19.75, *p* = 0.002) and dWMH (OR = 3.19; 95% CI 1.10–9.23, *p* = 0.032) were identified as independent predictors of END after adjusting for confounding factors (8). However, our study did not find any significant association between WMH and END. One possible explanation for this discrepancy is that the population in our study is younger than that of the referenced study, as WMH are known to be age-related imaging features; Furthermore, no other CSVD imaging biomarkers were included in their study, and the sample size was not sufficiently large to draw definitive conclusion.

Another study on intracranial branch atheromatous disease demonstrated that the culprit plaques in large parent arteries, rather than cerebral small vessel disease, contribute to early neurological deterioration in stroke patients with this condition (17). This finding is consistent with our study, in which nearly half of the subjects’ infarction mechanisms can be attributed to BABD. Additionally, our results suggest that vulnerable plaques in the basilar artery are independent predictor of END in multivariate regression analysis. Furthermore, a possible explanation for the negative correlation observed between CSVD and END in cases of isolated pontine infarction may be that the CSVD burden was primarily evaluated in the anterior circulation, which does not fully reflect the vascular autoregulatory capability in the posterior circulation.

### Other imaging predictors of END in isolated pontine infarction

Most previous studies on imaging biomarkers in isolated pontine infarction have focused on the topological morphological characteristics, including the infarct location, volume, and signal intensity on the Apparent Diffusion Coefficient (ADC). Ventral pontine infarcts (2), infarct volume (calculated as maximum length multiplied by thickness) (18), and the mean relative ADC (rADC) value within the ischemic lesion have been shown to be closely related to early neurological deterioration in patients with isolated pontine infarcts (19). We did not include these features in our study, as not all infarcts visible on imaging can be accurately reflected in the NIHSS score, particularly when dealing with the complex anatomy of the pons.

Another intriguing finding in our study is that anterior circulation vascular stenosis may serve as a potential predictor of END in this population, although it demonstrates only marginal statistical significance in both univariate and multivariate regression analyses. We hypothesize that patients with a greater atherosclerotic burden often experience some degree of dysfunction in cerebral autoregulation, however, this conclusion warrants further investigation.

### Advantages and limitations

Our study comprehensively investigated the association between the burden of cerebral small vessel disease, its independent features, and END in cases of isolated pontine infarction, despite the absence of positive findings. This research provides insight into the mechanisms underlying isolated pontine infarction. The results revealed that BABD is the most common mechanism associated with isolated pontine infarction, while basilar artery intraplaque hemorrhage is identified as an independent factor contributing to END in these patients. This study also contributes new knowledge regarding the imaging features that can predict END in the posterior circulation.

Some limitations should also be acknowledged here. First, the sample size in our study is not large enough to draw definitive conclusions, a larger sample study is needed in the future to validate our results. Second, we employed semi-quantitative scoring to evaluate the burden of CSVD, and no quantitative imaging features were utilized in this study, such as infarct volume, white matter hyperintensity volume, and perfusion index, among others. Finally, the cause-and-effect relationship could not be established due to the retrospective nature of the study, and some bias could possibly also have an effect on the conclusion.

## Conclusion

In conclusion, early neurological deterioration in isolated pontine infarction is not uncommon, the mechanisms underlying END and the related factors warrant further investigation. Our study demonstrated a negative correlation between cerebral small vessel disease and END in isolated pontine infarction. However, the presence of vulnerable plaques in the basilar artery as a predictor of END in these patients deserve additional attention.

## Data Availability

The raw data supporting the conclusions of this article will be made available by the authors, without undue reservation.

## References

[1] Yang H Liu H Zhang K Zong C Wang A Wang Y. Neuroimaging markers of early neurological deterioration in acute isolated pontine infarction. Neurol Sci. (2023) 44:3607–14. doi: 10.1007/s10072-023-06837-2, PMID: 37246178

[2] Vynckier J Maamari B Grunder L Goeldlin MB Meinel TR Kaesmacher J. Early neurologic deterioration in lacunar stroke. Neurology. (2021) 97:2661. doi: 10.1212/WNL.0000000000012661, PMID: 34400585

[3] Vemmos KN Spengos K Tsivgoulis G Manios E Zis V Vassilopoulos D. Aetiopathogenesis and long-term outcome of isolated pontine infarcts. J Neurol. (2005) 252:212–7. doi: 10.1007/s00415-005-0639-9, PMID: 15729529

[4] Wardlaw JM Smith EE Biessels GJ Cordonnier C Fazekas F Frayne R. Neuroimaging standards for research into small vessel disease and its contribution to ageing and neurodegeneration. Lancet Neurol. (2013) 12:822–38. doi: 10.1016/S1474-4422(13)70124-8, PMID: 23867200 PMC3714437

[5] Song TJ Kim J Song D Yoo J Lee HS Kim YJ. Total cerebral small-vessel disease score is associated with mortality during follow-up after acute ischemic stroke. J Clin Neurol. (2017) 13:187–95. doi: 10.3988/jcn.2017.13.2.187, PMID: 28406586 PMC5392462

[6] Lau KK Li L Schulz U Simoni M Chan KH Ho SL. Total small vessel disease score and risk of recurrent stroke. Neurology. (2017) 88:2260–7. doi: 10.1212/WNL.0000000000004042, PMID: 28515266 PMC5567324

[7] Chen Z Li W Sun W Xiao L Dai Q Cao Y. Correlation study between small vessel disease and early neurological deterioration in patients with mild/moderate acute ischemic stroke. Int J Neurosci. (2017) 127:579–85. doi: 10.1080/00207454.2016.1214825, PMID: 27430627

[8] Nam KW Lim JS Kang DW Lee YS Han MK Kwon HM. Severe white matter Hyperintensity is associated with early neurological deterioration in patients with isolated pontine infarction. Psychiatr Neurol. (2016) 76:117–22. doi: 10.1159/000448888, PMID: 27532619

[9] Liu Z Ma H Guo ZN Wang L Qu Y Fan L. Impaired dynamic cerebral autoregulation is associated with the severity of neuroimaging features of cerebral small vessel disease. CNS Drug Rev. (2022) 28:298–306. doi: 10.1111/cns.13778, PMID: 34894087 PMC8739047

[10] Staals J Makin SD Doubal FN Dennis MS Wardlaw JM. Stroke subtype, vascular risk factors, and total MRI brain small-vessel disease burden. Neurol Genet. (2014) 83:1228–34. doi: 10.1212/WNL.0000000000000837, PMID: 25165388 PMC4180484

[11] Fazekas F Chawluk JB Alavi A Hurtig HI Zimmerman RA. MR signal abnormalities at 1.5 T in Alzheimer's dementia and normal aging. Am J Roentgenol. (1987) 149:351–6. 3496763 10.2214/ajr.149.2.351

[12] Song X Zhao X Liebeskind DS Xu W Zhang J Wei C. Associations between systemic blood pressure parameters and intraplaque hemorrhage in symptomatic intracranial atherosclerosis: a high-resolution MRI-based study. Hypertens Res. (2020) 43:688–95. doi: 10.1038/s41440-020-0411-7, PMID: 32037397

[13] Wu L Li Y Ye Z Liu D Dai Z Zhu J. Site and mechanism of recurrent pontine infarction: a hospital-based follow-up study. Brain Sci. (2022) 12:520. doi: 10.3390/brainsci12050520, PMID: 35624909 PMC9138740

[14] Coutureau J Asselineau J Perez P Kuchcinski G Sagnier S Renou P. Cerebral small vessel disease MRI features do not improve the prediction of stroke outcome. Neurol Genet. (2021) 96:e527–37. doi: 10.1212/WNL.0000000000011208, PMID: 33184231

[15] Rensma SP van T Launer LJ Stehouwer C. Cerebral small vessel disease and risk of incident stroke, dementia and depression, and all-cause mortality: a systematic review and meta-analysis. Neurosci Biobehav Rev. (2018) 90:164–73. doi: 10.1016/j.neubiorev.2018.04.003, PMID: 29656031 PMC6123527

[16] Wang X Lyu J Meng Z Wu X Chen W Wang G. Small vessel disease burden predicts functional outcomes in patients with acute ischemic stroke using machine learning. CNS Drug Rev. (2023) 29:1024–33. doi: 10.1111/cns.14071, PMID: 36650639 PMC10018092

[17] Men X Hu M Guo Z Li Y Zheng L Wu R. Culprit plaques of large parent arteries, rather than cerebral small vessel disease, contribute to early neurological deterioration in stroke patients with intracranial branch atheromatous disease. Cerebrovas Dis. (2024) 53:88–97. doi: 10.1159/000530371, PMID: 36996763

[18] Li H Dai Y Wu H Luo L Wei L Zhou L. Predictors of early neurologic deterioration in acute pontine infarction. Stroke. (2020) 51:637–40. doi: 10.1161/STROKEAHA.119.027239, PMID: 31795900

[19] Oge DD Topcuoglu MA Arsava EM. Apparent diffusion coefficient signature of ischemic tissue predicts neurological progression in isolated pontine infarcts. Eur Stroke J. (2022) 7:66–70. doi: 10.1177/23969873211072956, PMID: 35300260 PMC8921789

